# *Cornus mas* L. Stones: A Valuable by-Product as an Ellagitannin Source with High Antioxidant Potential

**DOI:** 10.3390/molecules25204646

**Published:** 2020-10-12

**Authors:** Dominika Przybylska, Alicja Z. Kucharska, Iwona Cybulska, Tomasz Sozański, Narcyz Piórecki, Izabela Fecka

**Affiliations:** 1 Department of Fruit, Vegetable and Plant Nutraceutical Technology, Wrocław University of Environmental and Life Sciences, Chełmońskiego 37, 51-630 Wrocław, Poland; alicja.kucharska@upwr.edu.pl; 2 Earth and Life Institute, Université Catholique de Louvain, Croix du Sud 2, 1348 Louvain-la-Neuve, Belgium; iwona.cybulska@uclouvain.be; 3 Department of Pharmacology, Wrocław Medical University, Jana Mikulicza-Radeckiego 2, 50-345 Wrocław, Poland; tomasz.sozanski@umed.wroc.pl; 4 Arboretum and Institute of Physiography in Bolestraszyce, 37-700 Przemyśl, Poland; narcyz360@gmail.com; 5 Institute of Physical Culture Sciences, Medical College, University of Rzeszów, Towarnickiego 3, 35-959 Rzeszów, Poland; 6 Department of Pharmacognosy and Herbal Medicines, Wroclaw Medical University, Borowska 211 A, 50-556 Wrocław, Poland; izabela.fecka@umed.wroc.pl

**Keywords:** *Cornus mas* stones, UPLC-ESI-qTOF-MS/MS, gallotannins, ellagitannins, technological waste, bioactive compounds, antioxidants

## Abstract

The stone of *Cornus mas* L. remains the least known morphological part of this plant, whereas the fruit is appreciated for both consumption purposes and biological activity. The stone is considered to be a byproduct of fruit processing and very little is known about its phytochemical composition and biological properties. In this study, the complete qualitative determination of hydrolyzable tannins, their quantitative analysis, total polyphenolic content, and antioxidant properties of the stone of *C. mas* are presented for the first time. The 37 identified compounds included the following: various gallotannins (**11**), monomeric ellagitannins (**7**), dimeric ellagitannins (**10**), and trimeric ellagitannins (**7**). The presence of free gallic acid and ellagic acid was also reported. Our results demonstrate that *C. mas* stone is a source of various bioactive hydrolyzable tannins and shows high antioxidant activity which could allow potential utilization of this raw material for recovery of valuable pharmaceutical or nutraceutical substances. The principal novelty of our findings is that hydrolyzable tannins, unlike other polyphenols, have been earlier omitted in the evaluation of the biological activities of *C. mas*. Additionally, the potential recovery of these bioactive chemicals from the byproduct is in line with the ideas of green chemistry and sustainable production.

## 1. Introduction

Cornelian cherry (*Cornus mas* L.), which belongs to the Cornaceae family, is one of the two species from genus Cornus, which have been used in traditional ethnomedicine. It is native principally to Central and South Eastern Europe, while the second species, *Cornus officinalis* Torr. ex Dur., grows in Asia. These two species form a closely related phylogenetical pair [1]. They show numerous similarities of phytochemical profile and several differences, which are reflected by their therapeutic applications. Knowledge of the medicinal use of *C. mas* dates back to traditional medicine practices, for example, in Greece, Turkey, Slovakia, and China, where it served for prevention and treatment of gastrointestinal and circulatory disorders, diabetes, diarrhea, and flu. According to the literature reports, fruits were used most frequently, whereas leaves, flowers, and fruit stones were applied to a minor extent [2,3].

Cornelian cherry bears single-stone fruits of mostly dark-red color. Their shape is oval or spherical, and they have an average length of 1.00–2.22 cm and weight of 0.39–3.78 g [4,5]. Fruits are attractive for direct consumption and production of jams, juices, alcoholic beverages, and pickles which have been proven to be a rich source of health-promoting compounds, such as polyphenols (anthocyanins, flavonols, and phenolic acids), iridoids, terpenoids (ursolic acid), and vitamin C [2,6,7]. This fact probably justifies the traditional applications of cornelian cherry [2,8]. Recently, several in vitro and in vivo studies have confirmed antioxidant, anti-inflammatory, antidiabetic, hypolipidemic, anti-atherosclerotic, antimicrobial, and anticancer activity of the fruits [3,7,8,9,10,11,12,13,14].

Cornelian cherry stone forms 8.0–15.9% [5,14] or 5.7–11.0% [3] of total fruit weight and is considered to be a waste material after fruit processing. Exploitation of fruit stones (technological wastes) is not a new issue for the food industry. The conventional and emerging technologies applied for the valorization of technological wastes or for the recovery of nutraceuticals (e.g., fruit and vegetable antioxidants) have been reviewed previously [15,16,17]. One of the possible utilizations of discarded fruit stones is their conversion into biofuels. This solution was also proposed for cornelian cherry stones, to obtain bio-oil from hydrothermal liquefaction [18]. Similar uses can be found for the residual stones of cherry, plum, and peach [19,20,21,22].

In addition, fruit stones, as a morphological part, seem to be a promising raw material, in connection with bioactive phytochemicals. There are some papers from recent years which have discussed possible uses of fruit stones or ingredients from them for nutritional and therapeutic purposes [19,23]. The above represent some attempts to lower the food wastes generated at the processing plant. Re-utilization of such byproducts meets the sustainable consumption and production (SDG 12) goals of the United Nations (UN) 2030 Agenda for Sustainable Development, announced in 2015 [24]. 

The stone of cornelian cherry continues to be the least known part of this plant in terms of the composition and biological properties. According to the available literature, only fatty acids and minerals (Ca, K, P, Mg, Na, and Cu) have been reported in the stones of cornelian cherry [14,25]. Six fatty acids have been identified in the oil fraction of stones and, importantly, about 90% constituted unsaturated fatty acids. According to Kucharska et al. [14] and Vidrih et al. [25], the highest content, in the range of 64.8–75.0%, was noted for linoleic acid, followed by 15.0–22.9% for oleic acid, and 1.3–2.1% for linolenic acid. The remaining fatty acids were stearic, palmitic, and arachidic acid. Such fatty acids composition corresponds to that of commonly consumed vegetable oils from sunflower, corn, or pumpkin [25].

Very few reports concerning traditional curative uses of cornelian cherry stones state that oil from stones showed a healing effect for wounds, stomach ulcers, and colitis (Iran, Azerbaijan, Armenia, Georgia, and Turkey) [2]. According to another source, a mixture of stones and honey was ingested against diabetes [2]. Additionally, roasted cornelian cherry stones were used in traditional folk practice as a coffee substitute due to similar aroma, but specific recipes for this infusion are not available. Despite the above, beneficial activities of cornelian cherry stones have not been proven in modern scientific experiments as far as we know.

Hydrolyzable tannins are polyphenolic compounds which form complex molecular structures. They are esters built of some phenolic acids, for example, galloyl (gall), hexahydroxydiphenoyl (HHDP), valoneoyl (val), and a sugar moiety, usually d-glucose [26,27]. When subjected to hydrolysis, the HHDP group is released and converted into ellagic acid [27]. The presence of hydrolyzable tannins has been reported in certain fruits, for example, raspberries, blackberries, strawberries, and pomegranates, as well as in non-edible parts, i.e., leaves, roots, and seeds of raspberry, blackberry, strawberry, pomegranate, and some nuts [28]. In the case of the genus Cornus, hydrolyzable tannins have been previously identified by Okuda et al. [29] and Hatano et al. [30,31,32], in fruits of *C. officinalis*. Until recently, it was believed that tannins appeared only in *C. officinalis* and were perceived as the main difference between the two species. However, ellagic acid, a marker of hydrolyzable ellagitannins, was found in both *Cornus* species, and therefore it could possibly indicate the presence of ellagitannins in *C. mas* [5,7,27]. Moreover, Efenberger-Szmechtyk et al. [33], identified some ellagitannins in the leaves of *C. mas*. To our knowledge, there are no studies on the identification of tannins in *C. mas* stones and no works which have addressed their health promoting characteristics. For this reason, the aim of our research was to present the first complete determination of hydrolyzable tannins in cornelian cherry stones and analyze their antioxidant activity.

## 2. Results

### 2.1. Qualitative Identification by Means of UPLC-ESI-qTOF-MS/MS

The compounds were identified on the basis of UPLC retention time (*t_R_*), elution order, spectra UV-Vis (200–600 nm), MS, MS/MS fragmentation (acquired in negative mode), and by comparison with literature data when available. In our research, we determined 35 polyphenolic compounds from two groups, i.e., gallotannins and ellagitannins, including different isomers. Common MS/MS fragments for the given compounds were at *m*/*z* 169 and 125 from the gallic acid residue characteristic for galloyl esters, and a fragment ion of ellagic acid at *m/z* 301 from the HHDP esters, by comparison with the literature [34,35,36,37,38,39]. Additionally, free gallic acid (**2**) and ellagic acid (**33**) were present in the extract.

**Figure 1:**
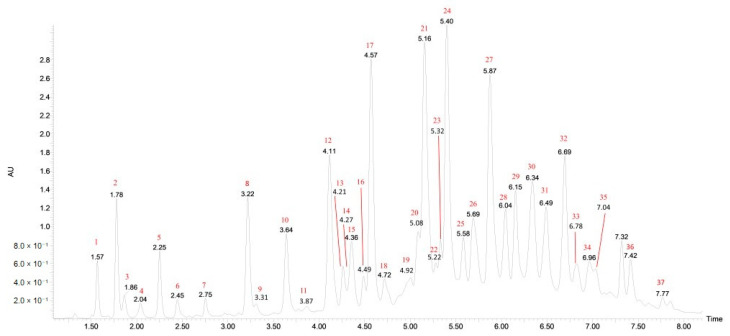
UPLC-PDA chromatogram of cornelian cherry stone extract at λ = 280 nm. Peak numbers refer to compounds listed in Table 1 and Table S1.

A typical UPLC chromatogram of the analyzed aqueous solution of ethanolic extract at 280 nm is shown in Figure 1 and the complete results of qualitative identification of the polyphenolic compounds of cornelian cherry stones are presented in Table 1 and Table S1.

Tandem mass spectrometry (UPLC-ESI-qTOF-MS/MS) allowed us to identify eleven compounds from the group of gallotannins and molecular weight 332–940 Da.

Compounds **1** and **5** gave a deprotonated pseudomolecular ion [M − H]^−^ at *m*/*z* 331.0639 and some fragment ions from gallic acid residue at *m*/*z* 169 [GA − H]^−^, and 125 (decarboxylated galloyl, −44 Da) characteristic for mono-*O*-galloyl-β-d-glucose. These isomers describe the simplest gallotannin included in our experiment. Compounds **4**, **7**, **9**, and **13** provided an [M − H]^−^ ion at 483.0763, which corresponded to the pseudomolecular ion of di-*O*-galloyl esters of glucose, and a fragment ion at *m*/*z* 331, after loss of one galloyl moiety (−152 Da). Thus, these were identified as four isomers of di-*O*-galloyl-β-d-glucose. Compound **16** showed the same [M − H]^−^ ion at *m*/*z* = 635.0872 as compound **19**, which indicated two isomers of tri-*O*-galloyl-β-d-glucose. These compounds showed the same fragment ion at *m*/*z* 465 after loss of one galloyl and one water molecule (−170 Da), and the ion at *m*/*z* 313 after loss of two galloyls and one water (−322 Da), which were equal to the dehydrated fragment of di-*O*-galloyl-β-d-glucose and mono-*O*-galloyl-β-d-glucose, respectively. Compound **32** gave a pseudomolecular ion [M − H]^−^ at *m*/*z* = 787.1022 and it was assigned to tetra-*O*-galloyl-β-d-glucose. The most complex derivatives of **1** were compounds **36** and **37**, which gave pseudomolecular ions [M − H]^−^ at *m*/*z* = 939.1080 and 939.1143, respectively, that corresponded to two isomers of penta-*O*-galloyl-β-d-glucose. Similarly, isomers of penta-*O*-galloyl-β-d-glucose liberated fragment ions through the loss of consecutive galloyl units and water molecules, and provided the ions at *m*/*z* 787, 769, 617, 465, 313, 295, 169, and 125 (Table S1).

**Figure 2:**
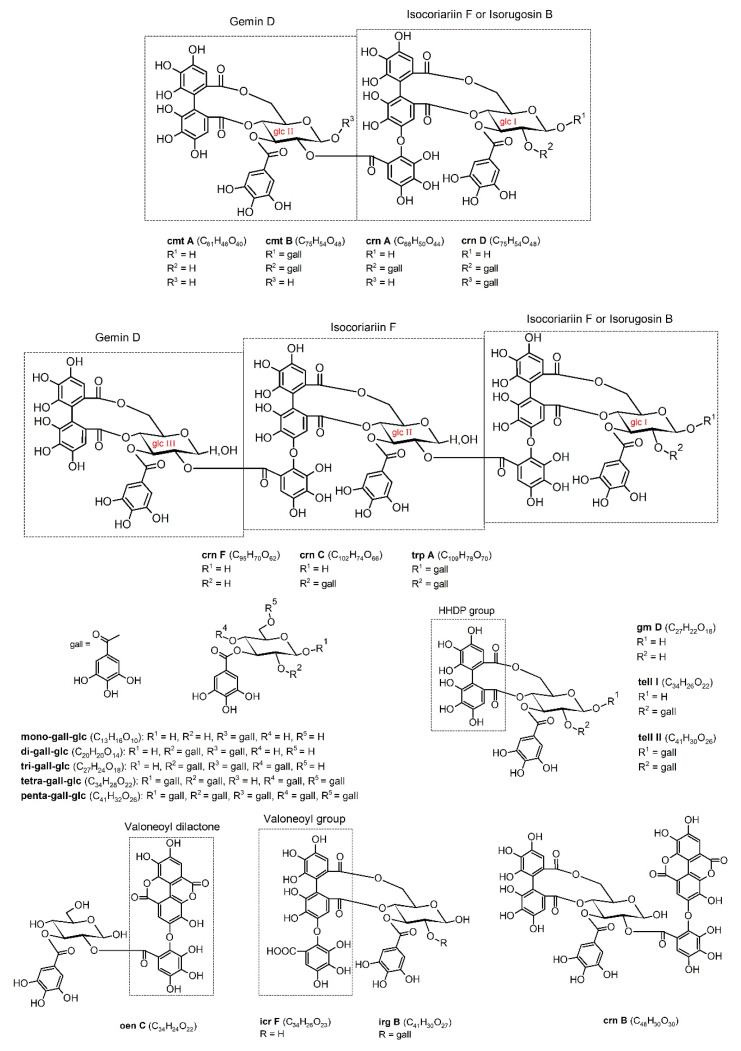
Chemical structures of the identified hydrolyzable tannins. Abbreviations: gall, galloyl; glc, β-d-glucose; gm D, gemin D; tell I, tellimagrandin I; tell II, tellimagrandin II; oen C, oenothein C; icr F, isocoriariin F; irg B, isorugosin B; crn B, cornusiin B; cmt A, camptothin A; cmt B, camptothin B; crn A, cornusiin A; crn D, cornusiin D; crn F, cornusiin F; crn C, cornusiin; trp A, trapanin A.

The collected data show that fragmentation of mono- to penta-galloyl derivatives results in the loss of subsequent galloyl moieties and water molecules, among other fragments, which are derived from the gallic acid residue (*m*/*z* 169) and decarboxylated gallic acid (*m*/*z* 125). Structures of the above gallotannins are shown in Figure 2. 

The second group of characterized compounds comprised ellagitannins. Among ellagitannins, we identified seven monomeric, ten dimeric, and seven trimeric compounds of molecular weight 634–2506 Da. Identified ellagitannins consist of a glucose core, galloyl and HHDP groups attached at the *O*-3 and *O*-4/*O*-6 of glucose, as well as valoneoyl or lactonized valoneoyl groups, which create *O*-2 and *O*-4/*O*-6 linkages between two or three glucose cores. 

Compounds **3** and **6** displayed the same pseudomolecular ion [M − H]^−^ at *m*/*z* = 633.0718, corresponding to the molecular formula of gemin D [29,31,37,38], the simplest among the ellagitannin molecules identified here, with the one group of HHDP and galloyl. Compound **11** had a pseudomolecular ion at *m*/*z* = 953.0872 and fragment ions at 909 (decarboxylated, −44 Da), 785, 783 (degalloylated and dehydrated, −170 Da), and 633 (galloyl-HHDP-glucose, e.g., gemin D), which indicated isorugosin B of the molecular weight of 954.0974 Da [28,35]. Compounds **14** and **20** showed an [M − H]^−^ ion at *m*/*z* = 785.0821, suggesting two isomeric forms of tellimagrandin I [31,37,38,39,40], which were derivatives of gemin D with an additional galloyl unit attached to the glucose core. Compound **31** gave a pseudomolecular ion [M − H]^−^ at *m/z* = 937.0892 corresponding to a molecular weight of 938.6629 Da of tellimagrandin II [32,38,40], ellagitannin with three galloyl units attached to glucose. Compound **22**, with [M − H]^−^ at *m*/*z* = 1085.0734 and fragment ions diminished by galloyl (*m*/*z* 933) and HHDP (*m*/*z* 783), was identified as cornusiin B, the monomer of the highest molecular weight (1086.7357 Da) [29,31,37]. 

Compounds **8**, **10, 17**, **23**–**25**, **28**–**30**, and **35** represented dimeric ellagitannins. Peaks **12**, **15**, **18**, **21**, **26**, **27,** and **34** represented trimeric ellagitannins. Among these groups, MS spectra for each compound, except **34**, displayed two typical pseudomolecular ions, [M − H]^−^ and [M − 2H]^−2^ [36], and in all cases, the doubly charged ion showed higher abundance.

Ellagitannins **8** and **10**, provided [M − 2H]^−2^ at *m/z* = 708.0688 and [M − H]^−^ at *m/z* = 1417.1549, which were identified as two anomers or other isomers of camptothin A, a dimer in which gemin D and isocoriariin F constitute monomers [30,33]. Compounds **17**, **23**, **24**, **25,** and **28** gave two ions, [M − 2H]^−2^ at *m*/*z* = 784.0729 and [M − H]^−^ at *m/z* = 1569.1556, which indicated five isomers of galloyl-camptothin A known as cornusiin A [29,31,33]. In addition, two isomers of cornusiin A (**17**, **23**) fragmented to the ion at *m*/*z* 1417, after the loss of galloyl, but all its isomers detached a fragment of monomeric ellagitannins such as gemin D (at *m*/*z* 633) and tellimagrandin I (at *m*/*z* 785), which indicated that the compound consisted of these structures, as well as a fragment (at *m*/*z* 783) of oenothein C or dehydrated isocoriariin F (from 802 − 18 = 784) [26,29,31], the ion at *m*/*z* 935 corresponding to the fragment formed after loss of HHDP, galloyl, and glucose (e.g., gemin D). Peaks **29** (*t_R_* = 6.15 min), **30** (*t_R_* = 6.34 min), and **35** (*t_R_* = 7.04 min), for ions [M − 2H]^−2^ at *m*/*z* = 860.0745 and [M − H]^−^ at *m*/*z* = 1721.1445, were tentatively identified as three positional isomers or anomers of either cornusiin D or camptothin B, which are galloyl esters of cornusiin A (additional galloyl in the structure) and have the same molecular weight but differ in the position of the one galloyl group, R^1^ vs. R^3^ at *O*-1 of glucose 1 or glucose 2 (Figure 2) [26,29,32,37]. The fragmentation of cornusiin D or camptothin B was identical to other dimeric ellagitannins with the valoneoyl bridge. 

Compounds **12**, **15**, **18,** and **21**, **26**, and **27** describe isomers of trimeric ellagitannins, cornusiin F and cornusiin C, respectively, both of which we identified in the extract at three spatial forms [31,33]. Compound **34** was trapanin A [41]. The molecular weights of isomers of cornusiin F, cornusiin C, and trapanin A were 2203.5389, 2355.6434 Da, and 2506.2544 Da, respectively. Compounds **12** and **15** gave doubly charged pseudomolecular ions at *m*/*z* = 1100.6101 and single-charge ions at *m*/*z* = 2201.1279. Similarly, compound **18** produced ions [M − 2H]^−2^ at 1101.1184 and [M − H]^–^ at 2201.1184. Two stereoisomers of cornusiin F (**12**, **15**) fragmented to ions at *m*/*z* = 2031, after the loss of one galloyl and one water molecule, and all of them displayed an ion at *m*/*z* 1247 diminished by isorugosin B [M − 954 − H]^−^. Figure 2 depicts the structures of the dimeric and trimeric ellagitannins. 

**Figure 3:**
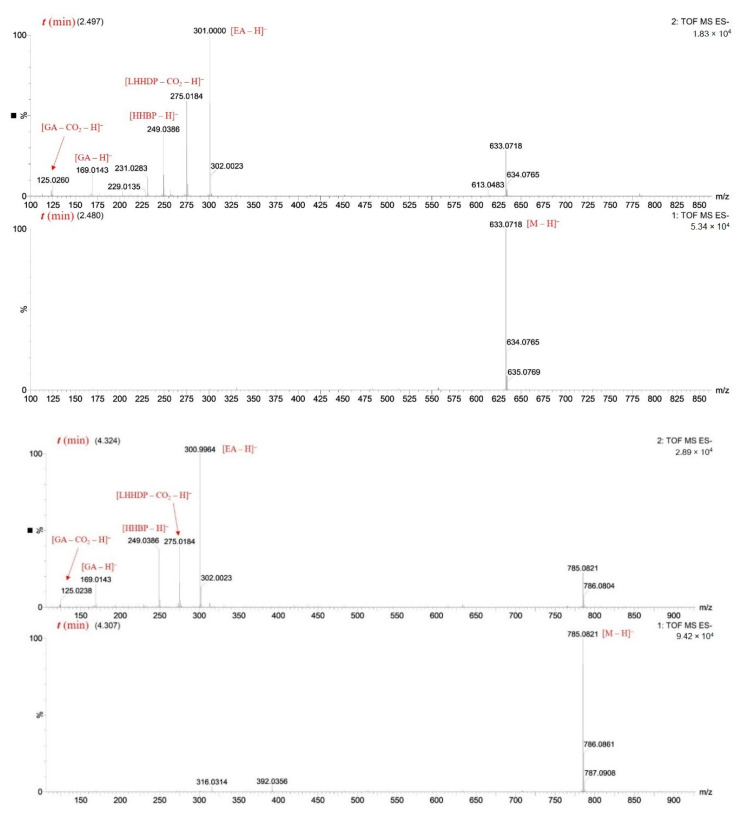

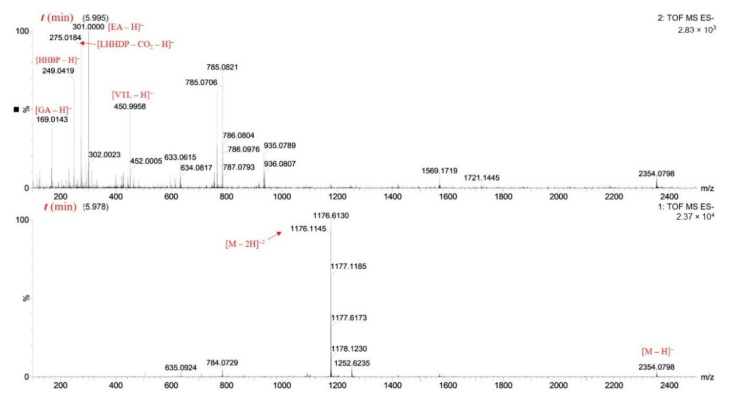
MS spectra of selected ellagitannins and products of their fragmentation (generated depsides). Abbreviations: GA, gallic acid; EA, ellagic acid; LHHDP, decarboxylated hexahydroxydiphenic acid monolactone; HHBP, 2,2’,3,3’,4,4’-hexahydroxybiphenyl; VTL, valoneic acid trilactone.

**Figure 4:**
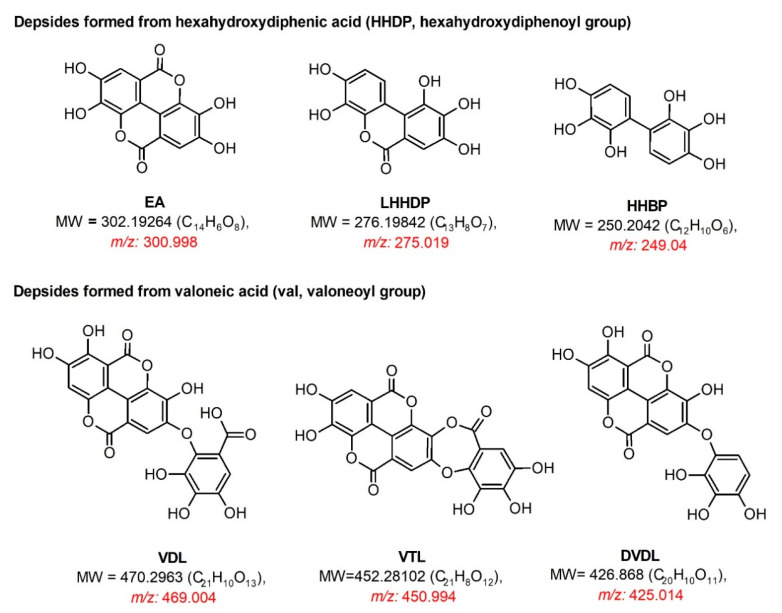
Products of the fragmentation of ellagitannins (depsides). Abbreviations: EA, ellagic acid; LHHDP, decarboxylated hexahydroxydiphenic acid monolactone; HHBP, 2,2′,3,3′,4,4′-hexahydroxybiphenyl; VDL, valoneic acid dilactone; VTL, valoneic acid trilactone; DVDL, decarboxylated valoneic acid dilactone; MW, molecular weight (Da).

As shown inTable S1, oligomeric ellagitannins from cornelian cherry stones during MS/MS experiments disconnected the fragment ions derived from their constituent monomers, for example, ions 953, 785, 783, 633, 331 and dehydrated monomers 765, 613, and 313. The detached HHDP group (−302 Da) was a source of ions at *m*/*z* 301, 275, and 249 derived from ellagic acid, and its degraded forms decarboxylated hexahydroxydiphenic acid monolactone (LHHDP) and 2,2′,3,3′,4,4′-hexahydroxydiphenyl (HHBP). MS/MS analysis also revealed the presence of other depsides, derived from a valoneoyl group, at *m*/*z* 451 (valoneoic acid trilactone, VTL) and 425 (decarboxylated valoneoic acid dilactone, DVDL) (Table S1). Although the fragment ion of valoneic acid dilactone (VDL) was not detected in the experiment, this structure occurred in oenothein C and cornusiin B (Figure 2), and the depsides VTL and DVDL were formed as its derivatives. When the suitable fragment ions appear on the MS spectrum, it indicates whether the compound contains HHDP or a valoneoyl unit. Selected MS spectra are shown in Figure 3 and structures of the formed depsides are shown in Figure 4**.**

According to Dong et al. [42], thirty tannins have been identified in the fruit of *C. officinalis*, which is considered to be a rich source of gallotannins and ellagitannins. The groups of Hatano and Okuda have broadly investigated the hydrolyzable tannins as follows: their isolation, identification of their structures, and occurrence of several isomers as a result of anomerization at the glucose cores of tannins in *C. officinalis* [26,27,28,29,30,31,32,43]. In the comparative review of two *Cornus* species, *C. mas* and *C. officinalis*, Czerwińska and Melzig [8] indicated that none of the tannins identified in the fruit of *C. officinalis* had been detected in the stones of *C. mas*.

The same gallotannins as determined here, except for penta-*O*-galloyl-β-d-glucose, were presented in review papers by Czerwińska and Melzig [8] and Dong et al. [42] as constituents of *C. officinalis* fruits.

Four isomers of camptothin A and four isomers of cornusiin A have been identified previously in the leaves of *Camptotheca acuminata* Decne. (Nyssaceae) and fruits of *C. officinalis* [29,31]. In the most recent paper, Efenberger-Szmechtyk et al. [33] reported the presence of four isomers of camptothin A and two isomers of cornusiin A in the leaves of *C. mas*. Herein, we identified five isomers of cornusiin A. In our study, the signal with the greatest abundance in the UPLC-PDA chromatogram was assigned to cornusiin C, and compound **21** seems to be the predominant isomer.

### 2.2. Quantitative Identification of Compounds

The content of hydrolyzable tannins was calculated from the regression equation for gallic acid. Free gallic and ellagic acid were identified on the basis of retention time, elution order, spectra UV-Vis, and compared to commercial standards. The tannins and gallic acid were identified at 280 nm and ellagic acid at 254 nm. Table 1 depicts the results of quantitative analysis. The total concentration of analyzed compounds (mean ± standard deviation) was 13,242.88 ± 37.07 mg/100 g of the extract, the total content of hydrolyzable tannins was 12,470.07 ± 66.53 mg/100 g of the extract and the contents of individual tannins ranged from 53.55 (camptothin A (**1**)) to 1354.73 (cornusiin C (**1**)) mg/100 g of the extract.

For the comparison, the content of hydrolyzable tannins and ellagic acid in strawberries ranged from 6.53 to 52.38 mg/100 g of fresh weight fw [34], the ellagitannin content in raspberries was 233.50 mg/100 g fw [44], and raspberry leaves contained 2.67–6.87% dw of tannins [45]. The data concerning ellagitannins in edible fruit stones is lacking and their content has been little discussed. However, Nowicka and Wojdyło [23] studied their presence in peach kernels and reported the presence of ellagic acid between 0.77 and 9.42 mg/100 g dw. Taken together, these data suggest that C. mas stones can be considered to be a novel rich source of ellagitannins, especially given that the stone of another fruit (peach) is a poor source of these compounds. 

### 2.3. Antioxidant Properties and Total Phenolic Content (TPC)

Since the hydrolyzable tannins belong to polyphenols, many of which are antioxidants, we investigated antioxidant properties of *C. mas* stones, rich in these components, using the spectrophotometric in vitro methods ABTS, FRAP, and DPPH, and total polyphenolic content assay (Table 2). 

So far, there were no papers which present antioxidant properties of *C. mas* stones, however, some authors claim strong antioxidant properties of fruit stones [23,46] and other sources of hydrolyzable tannins, for example, navy bean hull or strawberries [34,47,48]. According to Szajdek and Borowska [47], tannins are responsible for the antioxidant potency of navy bean seeds, which dominate among the polyphenols in the hull.

In our experiment, the value obtained in ABTS^•+^ free radical-scavenging assay was 255.99 ± 8.48 mmol TE/100 g. The reducing power measured in FRAP assay was 210.62 ± 5.45 mmol TE/100 g and the value obtained on the DPPH free radical-scavenging assay was 191.00 ± 0.04 mmol TE/100 g. The average content of compounds which reacted with the Folin–Ciocalteu reagent (TPC) was 11,466.53 ± 1971.76 mg GAE/100 g, while the TPC in the kernels of apricot obtained by other authors ranged from 10.60 to 209.4 mg GAE/100 g dw [46]. According to Nowicka and Wojdyło [23], the antioxidative potential (ABTS^•+^) of the peach kernel ranged from 2.19 to 27.20 mmol TE/100 g. Chen et al. [46] indicated that apricot kernels had significant free radicals-scavenging activities (ABTS^•+^ and DPPH), however, they expressed the values as median effective dose (EC_50_) and the results were difficult to compare. Nonetheless, our results indicate that stones of edible fruits, particularly the stones of cornelian cherry, are valuable raw materials in terms of antioxidant properties.

## 3. Discussion

The determined qualitative composition of hydrolyzable tannins in the stones of *C. mas* is comparable to that presented by other authors, although mostly in *C. officinalis*. A similar fragmentation pathway, characteristic for galloyl-*O*-glucoses and for ellagitannins, was obtained by other authors in previous studies concerning hydrolyzable tannins [34,35,38]. It is worth noting that the mass spectrometry and fragmentation studies of hydrolyzable tannins carried out so far explain mainly gallotannins and monomeric ellagitannins in *C. officinalis* or other plant genera, whereas data on oligomeric hydrolyzable tannins are limited, especially in *C. mas*.

According to available literature, ellagitannins detected in *Cornus sp*. demonstrate diverse biological activities. Lavoie et al. [49] reported that hydrolyzable tannins 1,2,3,6-tetra-*O*-galloyl-β-d-glucopyranose, 1,2,3,4,6-penta-*O*-galloyl-β-d-glucopyranose, tellimagrandin I, and tellimagrandin II possess antiviral properties against herpes simplex virus type 1 (HSV-1). Various hydrolyzable tannins, including gemin D, showed activity against human immunodeficiency virus (HIV), diminishing the HIV-induced cytopathogenic effect and HIV-specific antigen expression, and inhibiting binding of viruses to the target cells [50,51,52]. Interestingly, condensed tannins and other lower molecular weight polyphenols revealed no detectable anti-HIV activity [51,52]. Moreover, the ongoing SARS-CoV-2 pandemic crisis may increase the need for delivering new sources of antiviral compounds to the market. Growing consumers’ awareness of the diet-disease relationship raises their interest in fortified foods or dietary supplements for support of the immune system. A solid argument in favor of the use of hydrolyzable tannins is their plant origin, desirable to consumers. These premises point to the opportunity for natural sources of hydrolyzable tannins [53].

Tellimagrandin I significantly enhanced activity of β-lactam antibiotics against methicillin-resistant *Staphylococcus aureus* (MRSA) and decreased the minimum inhibitory concentrations (MICs) of oxacillin against the MRSA strains [26,54,55]. Tellimagrandin II showed antioxidant (DPPH) [50] and neuroprotective activity [42] and an inhibitory effect against yeast *Candida parapsilosis* ATCC 22,019 [56]. Tellimagrandin I, alongside the galloyl-*O*-glucoses, exhibited antioxidant activity in the DPPH test [57]. Berdowska et al. [58] evaluated the effect of the ellagitannins containing the HHDP group (tellimagrandin I, rugosin D and A, sanguiin H-6, agrimoniin, and pedunculagin), isolated from meadowsweet flowers (*Filipendulae ulmariae flos*, *Filipendula ulmaria* (L.) Maxim.), on the human breast cancer cell lines resistant to adriamycin (MCF-7/Adr) with reference to the wild type cell line (MCF-7/wt). The authors suggested that ellagitannins exhibited inhibitory activity towards the wild type cells and a stimulatory effect in the adriamycin-resistant cell model, in the case of all of the tested compounds, except sanguiin H-6. Moreover, penta-*O*-galloyl-β-d-glucose was found to have antioxidant activity (DPPH) and to inhibit skin carcinogenesis [50,59]. Ellagitannins containing galloyl and HHDP groups may be considered to be hepatoprotective agents diminishing liver injuries caused by free radicals. These groups are considered to be responsible for both antioxidant and hepatoprotective activity [60]. Cardullo et al. [61] studied the antidiabetic properties of selected ellagitannins and galloylated glucoses, including mono-*O*-galloyl-β-d-glucose, penta-*O*-galloyl-α-d-glucose, and penta-*O*-galloyl-β-d-glucose in the α-glucosidase and α-amylase inhibition tests. The tested compounds showed potent α-glucosidase inhibition. According to the authors, the gallotannins were more potent inhibitors towards α-glucosidase than ellagitannins. Strong inhibition of α-glucosidase was indicated as favorable regarding their future application in functional foods dedicated to preventing diabetes mellitus through the control of postprandial hyperglycemia.

In turn, Lee et al. [57] indicated potential activity of galloyl esters of glucose from the EtOAc-soluble fraction of *C. officinalis* stones against diabetic complications (e.g., cataracts). Other authors have reported that an 80% ethanolic extract from *C. officinalis* stones showed a stronger inhibitory effect on advanced glycation end-product (AGE) formation than the pericarp and fruits (pericarp with stones), and the most potent compounds found in the extract were 1,2,3-tri-*O*-galloyl-β-d-glucose, 1,2,6-tri-*O*-galloyl-β-d-glucose, 1,2,3,6-tetra-*O*-galloyl-β-d-glucose, 1,2,4,6-tetra-*O*-galloyl-β-d-glucose, 1,2,3,4,6-penta-*O*-galloyl-β-d-glucose, and tellimagrandin II [62]. Cornusiin A of *C. officinalis* fruits has been reported to exhibit an inhibitory effect on the reverse transcriptase of the RNA tumor virus and enhance host-mediated antitumor activity [29,32,63,64]. Park et al. [65] showed that cornusiin A exhibited an antiproliferative effect on androgen-sensitive human prostate cancer cells and this effect was mediated by apoptosis and S-phase cell cycle arrest. Another group presented the application of ellagitannins as functional ingredients in edible food packaging film, which improved its physical properties. At the same time, the formulation showed effective antioxidant activity and inhibitory activity towards *E. coli* and *S. aureus* strains [66]. Firstly, the edible substituents of artificial food packaging are less harmful to the environment and human health. Secondly, they prolong the shelf life of a product and even enhance its nutritional value.

Up to now, tannins have not been considered to be among the target bioactive compounds isolated from the species *C. mas*. Our study broadens the scope of knowledge about cornelian cherry stones’ chemical composition, as no previous papers mentioned either the presence of hydrolyzable tannins in this morphological part or their fragmentation pathways. Interestingly, the rich mixture of ellagitannins in cornelian cherry stones and their relatively high content could decide on their future exploitation, and therefore turn this technological waste into valuable secondary raw material. 

To summarize, the novelty of this study includes the innovative approach to discarded *C. mas* stones, as well as to hydrolyzable tannins from this species. These are undervalued among bioactive plant polyphenols although, as indicated above, they exhibit numerous biological activities which should be explored further.

## 4. Materials and Methods 

### 4.1. Reagents and Standards

All reagents and organic solvents were of analytical grade. Acetonitrile and 98–100% formic acid were acquired from Merck (Darmstadt, Germany). The water was glass distilled and deionized. Authentic standards of ellagic acid (EA) and gallic acid (GA) were purchased from Extrasynthese (Genay, France); 1,1-diphenyl-2-picrylhydrazyl (DPPH) ferrous chloride, tripyridyltriazine (TPTZ), kaliumperoxodisulfat, 2,2’-azino-bis(3-ethylbenzthiazoline-6-sulphonic acid) (ABTS), and 6-hydroxy-2,5,7,8-tetramethylchroman-2-carboxylic acid (Trolox) were obtained from Sigma Chemical Co. (Steinheim, Germany); and Folin–Ciocalteu reagent, methanol, acetonitrile, formic acid, and hydrochloric acid were obtained from POCh (Gliwice, Poland).

### 4.2. Raw Material

Cornelian cherry (*Cornus mas* L.) stones of the cultivar “Kostia” originated from the Arboretum in Bolestraszyce, Przemyśl, Poland. The relevant voucher specimen (“Kostia”–BDPA 14131) was deposited at the Herbarium of Arboretum in Bolestraszyce, Poland. After manual separation from fruits, stones were air-dried at room temperature, and after milling (IKA A11 Basic, Staufen, Germany), immediately analyzed.

### 4.3. Sample Preparation

Total solids (TS) and content of ashes (Ash) were determined in the raw material (milled stones) prior to the extraction, to give mean values of TS = 92.98% (*n* = 2) and Ash 1.04%, (*n* = 2). Milled stones were subjected to Soxhlet extraction in the sample-to-solvent ratio 1:30 (m/V). The solvent used for extraction was ethanol (absolute). Homogeneous plant material (5 g) was inserted into previously ignited (575 °C/12 h) and weighed ceramic thimbles. Extraction was carried out until the liquid in the Soxhlet chamber became transparent (3 h). Extraction was done in duplicate. Subsequently, the obtained ethanolic extract was concentrated by a vacuum rotary evaporator (Rotavapor R, Büchi, Flawil, Switzerland) and dried in a conventional dryer (40 °C) to obtain a powder. The extraction yielded in the mean value of 0.464 g of powder (8.63% dw of milled stones). In order to prepare the analyzed aqueous solution, powdered ethanolic extracts were re-dissolved in distilled water (20 mg/mL), vortexed for 15 s, sonicated in an ultrasonic water bath for 15 min at room temperature, and centrifuged (13,900 rpm/5 min). The supernatant was filtered through a PTFE 0.22 µm (qualitative analysis) and a PTFE 0.45 µm membrane (quantitative analysis and antioxidant assays) (Millex Samplicity Filter, Merck, Darmstadt, Germany).

### 4.4. Qualitative Identification by UPLC-ESI-qTOF-MS/MS

The method was previously described by Wyspiańska et al. [67]. Identification of compounds was performed using the Acquity ultra-performance liquid chromatography (UPLC) system, coupled with a quadrupole-time of flight (Q-TOF) MS instrument (UPLC/Synapt Q-TOF MS, Waters Corp., Milford, MA, USA), with an electrospray ionization (ESI) source. Separation was achieved on an Acquity UPLC BEH C18 column (100 × 2.1 mm i.d., 1.7 µm; Waters Corp., Milford, MA, USA). The mobile phase was a mixture of 2.0% aq. formic acid *v*/*v* (A) and acetonitrile (B). The gradient program was as follows: initial conditions, 1% B in A; 12 min, 25% B in A; 12.5 min, 100% B; 13.5 min, 1% B in A. The flow rate was 0.45 mL/min, and the injection volume was 5 µL. The column was operated at 30 °C. UV-Vis absorption spectra were recorded online during UPLC analysis, and the spectral measurements were made in the wavelength range of 200–600 nm, in steps of 2 nm. The major operating parameters for the Q-TOF MS were set as follows: capillary voltage 2.0 kV, cone voltage 40 V, cone gas flow of 11 L/h, collision energy 28–30 eV, source temperature 100 °C, desolvation temperature 250 °C, collision gas, argon; desolvation gas (nitrogen) flow rate, 600 L/h; data acquisition range, *m*/*z* 100–2500 Da. The compounds were monitored at 280 nm and explored in the negative mode before and after fragmentation. The data were collected with Mass-Lynx V 4.1 software (Waters Corp., Milford, MA, USA).

### 4.5. Quantitative Determination of Phenolic Compounds by HPLC-DAD

The HPLC analysis was performed according to Nowicka et al. [34] using a Dionex (Germering, Germany) system equipped with diode array detector Ultimate 3000, quaternary pump LPG-3400A, autosampler EWPS-3000SI, thermostated column compartment TCC-3000SD, and controlled by Chromeleon v.6.8 software. Separation was achieved using a Hypersil GOLD C18-column (250 × 4.6 mm, 5 μm; Thermo Fisher Scientific Inc., UK). The following mixtures were used as eluents: C, water-FA (98.5:1.5, *v/v*) and D, acetonitrile-FA (98.5:1.5, *v*/*v*). The gradient profile was as follows: initial conditions 100% C, 30 min; 30% D, 33 min; 70% D, 45 min; 70% D in C, 48 min; 100% D, 55–60 min; 100% C. The flow rate of the mobile phase was 1.2 mL/min, and the injection volume was 20 μL. The column was operated at 22 °C. The UV-Vis measurements were made in the wavelength range of 200–600 nm in steps of 2 nm. Gallotannins and ellagitannins were detected at 280 nm and quantified using linear regression equations based on an external standard of GA as mg of gallic acid equivalent per 100 g of the dried extract. Ellagic acid was detected at 254 nm and quantified using linear regression equations based on an external standard of EA, as mg of ellagic acid equivalent per 100 g of the extract. Results are provided as the mean ± standard deviation of two replications and expressed as milligrams per 100 g of the extract.

### 4.6. Total Phenolic Content and Antioxidant Activity

#### 4.6.1. Total Phenolic Content

Total phenolic content (TPC) assay was based on the method of Gao et al. [68], with slight modifications. Diluted stones extract (5 µL) was placed in each well of a 96-well plate and mixed with 10 µL of Folin–Ciocalteu reagent, 100 µL of H_2_O and 50 µL of 10% sodium carbonate, the mixture was shaken automatically for 30 s. Total polyphenols were determined after 1 h of incubation at room temperature in the dark. The absorbance of the resulting blue color was measured at 765 nm. The standard curve was prepared using different concentrations of gallic acid. The results were calculated as mg of gallic acid equivalent (GAE) per 100 g of the extract. The results were expressed as the mean ± standard deviations of four replications.

#### 4.6.2. ABTS, FRAP, and DPPH Assays

ABTS^•+^ (2,2’-azino-bis (3-ethyl benzothiazoline-6-sulfonic acid) assay was based on the method of Re et al. [69] with slight modifications. Briefly, ABTS radical cation is generated by reacting 7 mmol ABTS^•+^ and 2.45 mmol potassium persulfate via incubation at room temperature (23 °C) in the dark for 12–16 h. The ABTS^•+^ solution was diluted with an absorbance of 1.000 ± 0.200 at 734 nm. Then, 10 µL of diluted stones extract was placed in each well of a 96-well plate in four replications and 200 µL of prepared ABTS^•+^ solution was added automatically to the wells. The mixture was shaken at room temperature and the absorbance reading was taken 6 min after at 734 nm.

Ferric reducing antioxidant power (FRAP) assay was based on the method of Benzie and Strain [70] with slight modifications. Briefly, the FRAP reagent was prepared by mixing 50 mL of acetate buffer (300 M, pH 3.6), a solution of 0.0156 g 2,4,6-Tris(2-pyridyl)-s-triazine (TPTZ) reagent in 5 mL of 40 mmol HCl, and a solution of 0.02703 g ferric chloride in 5 mL H_2_O at 10:1:1 (*v*/*v*/*v*). Then, 10 µL of diluted stones extracts was placed in each well of a 96-well plate in four replications and 200 µL of prepared FRAP solution was added automatically to the wells. The mixture was shaken automatically for 30 s and the absorbance reading was taken 10 min after at 593 nm.

The DPPH free radical scavenging capacity of fruits extracts was measured from bleaching of the purple color of (2.2-diphenyl-1-picrylhydrazyl) based on the method of Yen and Chen [71], with some modifications. The DPPH ethanolic solution was diluted with an absorbance of 1.000 ± 0.200 at 517 nm. Then, 10 µL of diluted stones extracts was placed in each well of a 96-well plate in four replications and 200 µL of prepared DPPH solution was added automatically to the wells. The mixture was shaken, and the absorbance reading was taken 10 min after at 517 nm.

All measurements were recorded on a microplate reader Synergy H1 (BioTek, Winooski, VT, USA). The standard curve was prepared using different concentrations of Trolox. The results are expressed as Trolox equivalents (TE) per 100 g of the extract (mmol TE/100 g), the values represent the mean ± standard deviation. 

### 4.7. Chemical Structures

The chemical structures of compounds were prepared using the ACD/ChemSketch 2018.1.1 Freeware, Advanced Chemistry Development, Inc. (Toronto, ON, Canada).

## 5. Conclusions

In this paper we presented the first detailed identification of a total of 37 compounds of the stones of *Cornus mas* L., including gallotannins, ellagitannins, gallic acid, and ellagic acid. Many previous articles dedicated to *C. mas* omitted hydrolyzable tannins, especially their nutritional and health-promoting properties, given that tannins from plants were perceived as antinutrients and astringent agents. However, some of the gallotannins and ellagitannins have previously been proven t have beneficial properties, such as antioxidant, antidiabetic, antiphlogistic, antibacterial, hepatoprotective, antiviral, neuroprotective, and cancer preventing. This study contributes to the characterization of cornelian cherry stones and to overall knowledge about the genus Cornus. We showed that cornelian cherry stones, traditionally used for healing purposes, contain a number of different hydrolyzable tannins and have significant antioxidant properties. Our findings could justify the reuse of this technological waste for the recovery of bioactive substances. The extracts or specific isolated hydrolyzable tannins could be used as ingredients of functional foods or applied for therapeutic purposes. The perspectives for further research on phytochemicals from this raw material, including in the context of tannins, should involve the optimization of extraction, evaluation of the stability under processing and storage conditions, and last but not least, advanced assessment of the biological properties.

## Figures and Tables

**Table 1:** UPLC-ESI-qTOF-MS/MS and HPLC-DAD identification of hydrolyzable tannins in the extract of cornelian cherry stones.

Peak No.	*t_R_* (min)	λ_max_ (nm)	MW (Da)	MS^1^ [M − H]^–^ (*m/z*)	Quantity (mg/100 g of Extract)	Compound Name (Isomer)
					Mean ± SD	
1	1.57	215, 277	332.0743	331.0639 [M − H]^−^ 663.1382 [2M – H]^−^	256.29 ± 0.48	Mono-*O*-galloyl-β-d-glucose (**1**)
2	1.78	221, 270	170.0215	169.0143 [M − H]^−^	430.16 ± 2.77	Gallic acid
3	1.86	215, 265	634.0806	633.0718 [M − H]^−^	62.01 ± 6.45	Gemin D (**1**)
4	2.04	214, 272	484.0853	483.0763 [M – H]^−^	86.23 ± 0.41	Di-*O*-galloyl-β-d-glucose (**1**)
5	2.25	215, 277	332.0743	331.0639 [M − H]^−^	225.97 ± 8.22	Mono-*O*-galloyl-β-d-glucose (**2**)
6	2.45	215, 265	634.0806	633.0718 [M − H]^−^	399.91 ± 11.42	Gemin D (**2**)
7	2.75	214, 272	484.0853	483.0763 [M – H]^−^	33.49 ± 0.39	Di-*O*-galloyl-β-d-glucose (**2**)
8	3.22	222, 264	1418.1565	1417.1549 [M − H]^−^ 708.0688 [M – 2H]^−2^	53.55 ± 0.28	Camptothin A (**1**)
9	3.31	214, 272	484.0853	483.0763 [M – H]^−^	343.60 ± 1.38	Di-*O*-galloyl-β-d-glucose (**3**)
10	3.64	222, 264	1418.1565	1417.1549 [M − H]^−^ 708.0688 [M – 2H]^−2^	52.92 ± 0.64	Camptothin A (**2**)
11	3.87	214, 271	954.0974	953.0919 [M − H]^−^	591.55 ± 8.74	Isorugosin B
12	4.11	225, 266	2202.2325	2201.1279 [M – H]^−^ 1100.6101 [M − 2H]^−2^	460.31 ± 5.98	Cornusiin F (**1**)
13	4.21	214, 272	484.0853	483.0763 [M – H]^−^	61.05 ± 0.16	Di-*O*-galloyl-β-d-glucose (**4**)
14	4.27	218, 267	786.0916	785.0821 [M − H]^−^	226.94 ± 0.11	Tellimagrandin I (**1**)
15	4.36	225, 266	2202.2325	2201.1279 [M – H]^−^ 1100.6101 [M − 2H]^−2^	1186.56 ± 2.60	Cornusiin F (**2**)
16	4.49	215, 276	636.0963	1271.1876 [2M – H]^−^ 635.0872 [M – H]^−^	247.98 ± 32.96	Tri-*O*-galloyl-β-d-glucose (**1**)
17	4.57	232, 267	1570.1675	1569.1556 [M – 2H]^−^ 784.0729 [M – 2H]^–2^	123.15 ± 0.83	Cornusiin A (**1**)
18	4.72	225, 266	2202.2325	2201.1184 [M − H]^−^ 1100.6033 [M – 2H]^−2^	1336.99 ± 6.70	Cornusiin F (**3**)
19	4.92	215, 270	636.0963	1271.1949 [2M – H]^–^ 635.0872 [M − H]^−^	230.01 ± 0.01	Tri-*O*-galloyl-β-d-glucose (**2**)
20	5.08	218, 267	786.0916	785.0821 [M − H]^−^	353.08 ± 4.58	Tellimagrandin I (**2**)
21	5.16	232, 262	2354.2434	2353.0769 [M − H]^−^ 1176.1075 [M − 2H]^−2^	1354.73 ± 47.29	Cornusiin C (**1**)
22	5.22	219, 258	1086.0822	1085.0734 [M – H]^−^	211.59 ± 18.72	Cornusiin B
23	5.32	216, 275	1570.1675	1569.1556 [M − 2H]^−^ 784.0729 [M − 2H]^−2^	569.16 ± 19.64	Cornusiin A (**2**)
24	5.40	232, 262	1570.1675	1569.1556 [M – 2H]^−^ 784.0729 [M – 2H]^−2^	1115.76 ± 99.63	Cornusiin A (**3**)
25	5.58	218, 270	1570.1675	1569.1719 [M − 2H]^−^ 784.0729 [M − 2H]^−2^	181.21 ± 12.01	Cornusiin A (**4**)
26	5.69	221, 267	2354.2434	2353.0798 [M − H]^−^ 1176.1145 [M − 2H]^−2^	155.74 ± 12.44	Cornusiin C (**2**)
27	5.87	230, 268	2354.2434	2353.0798 [M − H]^−^ 1176.1145 [M − 2H]^−2^	304.03 ± 2.34	Cornusiin C (**3**)
28	6.04	221, 268	1570.1617	1569.1556 [M – H]^−^ 784.0729 [M – 2H]^−2^	560.56 ± 3.44	Cornusiin A (**5**)
29	6.15	222, 272	1722.1785	1721.1445 [M – H]^−^ 860.0745 [M – 2H]^−2^	391.86 ± 5.46	Cornusiin D or Camptothin B (**1**)
30	6.34	222, 271	1722.1785	1721.1445 [M – H]^−^ 860.0745 [M – 2H]^–2^	441.18 ± 5.16	Cornusiin D or Camptothin B (**2**)
31	6.49	221, 271	938.1025	937.0892 [M – H]^−^	168.79 ± 1.11	Tellimagrandin II
32	6.69	222, 275	788.1072	787.1022 [M – H]^−^	166.31 ± 3.91	Tetra-*O*-galloyl-β-d-glucose
33	6.78	254, 360	302.0063	300.9964 [M – H]^–^	342.65 ± 26.69	Ellagic acid
34	6.96	215, 272	2506.2544	1252.1165 [M – 2H]^–2^	84.77 ± 20.49	Trapanin A (β or α)
35	7.04	215, 271	1722.1785	1721.1700 [M – H]^–^ 860.0745 [M –2H]^–2^	108.11 ± 24.18	Cornusiin D or Camptothin B (**3**)
36	7.42	216, 277	940.1182	939.1080 [M – H]^–^	250.34 ± 8.59	Penta-*O*-galloyl-β-d-glucose (**1**)
37	7.77	216, 277	940.1182	939.1143 [M – H]^–^	74.36 ± 0.21	Penta-*O*-galloyl-β-d-glucose (**2**)

*t_R_*, retention time; λ_max_, maximum absorbance; MW, molecular weight (monoisotopic mass); MS^1^, the first mass spectrum (pseudomolecular ions); cmp A, camptothin A; crn B, cornusiin B; DVDL, decarboxylated valoneic acid dilactone; EA, ellagic acid; GA, gallic acid; gall, galloyl; glc, β-d-glucose; gm D, gemin D; HHBP, hexahydroxybiphenyl; HHDP, hexahydroxydiphenoyl; icr F, isocoriariin F; irg B, isorugosin B; LHHDP, hexahydroxydiphenic acid monolactone; oen C, oenothein C; tell I, tellimagrandin I; tell II, tellimagrandin II; VTL, valoneic acid trilactone.

**Table 2:** The results of antioxidant activity and total phenolic content assays of the extract of stones.

Antioxidant Activity			Total Phenolic Content
ABTS	FRAP	DPPH	
(mmol Tx/100 g)	(mmol Tx/100 g0	(mmol Tx/100 g)	(mg GAE/100 g)
255.99 ± 8.48	210.62 ± 5.45	191.00 ± 0.04	11,466.53 ± 1971.76

## Supplementary Materials

The following is available online. Table S1: UPLC-ESI-qTOF-MS/MS and HPLC-DAD identification of hydrolyzable tannins in the extract of cornelian cherry stones–other ions.
