# Supplementary material for: Cornus mas L. Stones: A Valuable by-Product as an Ellagitannin Source with High Antioxidant Potential
Source: Molecules. 2020 Oct 12;25(20):4646. doi: 10.3390/molecules25204646 (PMC7587210; doi:10.3390/molecules25204646)
Supplement: Supplementary file 1 [file molecules-25-04646-s001.pdf]

Table S1. UPLC-ESI-qTOF-MS/MS and HPLC-DAD identification of hydrolyzable tannins in the extract of cornelian cherry stones – other ions.

Peak No.	t_R (min)	MS² other ions (*m*/*z*)	Compound name (isomer)
1	1.57	169 [GA – H]⁻, 125 [GA – CO_2 (– 44) – H]⁻	Mono-*O*-galloyl-*β*-D-glucose (1)
2	1.78	125 [GA – CO_2 (– 44) – H]⁻	Gallic acid
3	1.86	301 [EA – H]⁻, 275 [LHHDP – CO_2 (– 44) – H]⁻, 249 [HHBP – H]⁻, 169 [GA – H]⁻, 125 [GA – CO_2 (– 44) – H]⁻	Gemin D (1)
4	2.04	331 [M – gall (– 152) – H]⁻, 169 [GA – H]⁻, 125 [GA – CO_2 (– 44) – H]⁻	Di-*O*-galloyl-*β*-D-glucose (1)
5	2.25	169 [GA – H]⁻, 125 [GA – CO_2 (– 44) – H]⁻	Mono-*O*-galloyl-*β*-D-glucose (2)
6	2.45	301 [EA – H]⁻, 275 [LHHDP – CO_2 (– 44) – H]⁻, 249 [HHBP – H]⁻, 169 [GA – H]⁻, 125 [GA – CO_2 (– 44) – H]⁻	Gemin D (2)
7	2.75	331 [M – gall (– 152) – H]⁻, 169 [GA – H]⁻, 125 [GA – CO_2 (– 44) – H]⁻	Di-*O*-galloyl-*β*-D-glucose (2)
8	3.22	1247 [M – gall – H_2O (– 170) – H]⁻, 1115 [M – HHDP (– 302) – H]⁻, 783 [oen C/M – HHDP – gall – glc = gm D (– 634) – H]⁻, 765 [oen C – H_2O (– 18) – H]⁻, 633 [gall-HHDP-glc = gm D – H]⁻, 613 [oen C – gall – H_2O (– 170) – H]⁻, 451 [VTL – H]⁻, 425 [DVDL – H]⁻, 331 [gall-glc – H]⁻, 313 [gall-glc – H_2O (– 18) – H]⁻, 301 [EA – H]⁻, 275 [LHHDP – CO_2 (– 44) – H]⁻, 249 [HHBP – H]⁻, 169 [GA – H]⁻, 125 [GA – CO_2 (– 44) – H]⁻	Camptothin A (1)
9	3.31	331 [M-gall (– 152) – H]⁻, 169 [GA – H]⁻, 125 [GA – CO_2 (– 44) – H]⁻	Di-O-galloyl-*β*-D-glucose (3)
10	3.64	1247 [M – gall – H_2O (– 170) – H]⁻, 1115 [M – HHDP (– 302) – H]⁻, 783 [oen C = M – HHDP – gall – glc = gm D (– 634) – H]⁻, 765 [oen C – H_2O (– 18) – H]⁻, 633 [gall-HHDP-glc = gm D – H]⁻, 613 [oen C – gall – H_2O (– 170) – H]⁻, 451 [VTL – H]⁻, 425 [DVDL – H]⁻, 331 [gall-glc – H]⁻, 313 [gall-glc – H_2O (– 18) – H]⁻, 301 [EA – H]⁻, 275 [LHHDP – CO_2 (– 44) – H]⁻, 249 [HHBP – H]⁻, 169 [GA – H]⁻, 125 [GA – CO_2 (– 44) – H]⁻	Camptothin A (2)
11	3.87	909 [M – CO_2 (– 44) – H]⁻, 785 [tell I – H]⁻, 783 [M – gall – H_2O – H]⁻, 633 [gall-HHDP-glc = gm D – H]⁻, 451 [VTL – H]⁻, 425 [DVDL – H]⁻, 301 [EA – H]⁻, 275 [LHHDP – CO_2 (– 44) – H]⁻, 249 [HHBP – H]⁻, 169 [GA – H]⁻, 125 [GA – CO_2 (– 44) – H]⁻	Isorugosin B
12	4.11	2031 [M – gall – H_2O (– 170) –H]⁻, 1247 [M – irg B (– 954) – H]⁻, 785 [tell I – H]⁻, 783 [oen C – H]⁻, 765 [oen C – H_2O (– 18) – H]⁻, 633 [gall-HHDP-glc = gm D – H]⁻, 613 [oen C – gall – H_2O (– 170) – H]⁻, 483 [di-gall-glc – H]⁻, 451 [VTL – H]⁻, 425 [DVDL – H]⁻, 331 [gall-glc – H]⁻, 313 [gall-glc – H_2O (– 18) – H]⁻, 301 [EA – H]⁻, 275 [LHHDP – CO_2 (– 44) – H]⁻, 249 [HHBP – H]⁻, 169 [GA – H]⁻, 125 [GA – CO_2 (– 44) – H]⁻	Cornusiin F (1)
13	4.21	331 [M – gall (–152) – H]⁻, 169 [GA – H]⁻, 125 [GA – CO_2 (– 44) – H]⁻	Di-*O*-galloyl-*β*-D-glucose (4)
14	4.27	301 [EA – H]⁻, 275 [LHHDP – CO_2 (– 44) – H]⁻, 249 [HHBP – H]⁻, 169 [GA – H]⁻, 125 [GA – CO2 (– 44) – H]⁻	Tellimagrandin I (1)
15	4.36	2031 [M – gall – H_2O (– 170) – H]⁻, 1247 [M – irg B (– 954) – H]⁻, 785 [tell I – H]⁻, 783 [oen C – H]⁻, 765 [oen C – H_2O (– 18) – H]⁻ 633 [gall-HHDP-glc = gm D – H]⁻, 613 [oen C – gall – H_2O (– 170) – H]⁻, 483 [di-gall-glc – H]⁻, 451 [VTL – H]⁻, 425 [DVDL – H]⁻, 331 [gall-glc – H]⁻, 313 [gall-glc – H_2O (– 18) – H]⁻, 301 [EA – H]⁻, 275 [LHHDP – CO_2 (– 44) – H]⁻, 249 [HHBP – H]⁻, 169 [GA – H]⁻, 125 [GA – CO_2 (– 44) – H]⁻	Cornusiin F (2)

16	4.49	465 [M – gall – H_2O (– 170) – H]$^-$, 313 [M – 2 x gall – H_2O (– 322) – H]$^-$, 169 [GA – H]$^-$, 125 [GA – CO_2 (– 44) – H]$^-$	Tri-*O*-galloyl-β-D-glucose (1)
17	4.57	1417 [cmp A = M – gall (– 152) – H]$^-$, 935 [M – HHDP – gall – glc = gm D (– 634) – H]$^-$, 785 [tell I – H = icr F – 18 – H]$^-$, 783 [oen C = M – HHDP – 2 x gall – glc (– 786) – H]$^-$, 765 [oen C – H_2O (– 18) – H]$^-$, 633 [gall-HHDP-glc = gm D – H]$^-$, 451 [VTL – H]$^-$, 425 [DVDL – H]$^-$, 313 [gall-glc – H_2O (– 18) – H]$^-$, 301 [EA – H]$^-$, 275 [LHHDP – CO_2 (– 44) – H]$^-$, 249 [HHBP – H]$^-$, 169 [GA – H]$^-$, 125 [GA – CO_2 (– 44) – H]$^-$	Cornusiin A (1)
18	4.72	1247 [M – irg B (– 954) – H]$^-$, 785 [tell I – H]$^-$, 783 [oen C – H]$^-$, 765 [oen C – H_2O (– 18) – H]$^-$, 633 [gall-HHDP-glc = gm D – H]$^-$, 613 [oen C – gall – H_2O (– 170) – H]$^-$, 483 [di-gall-glc – H]$^-$, 451 [VTL – H]$^-$, 425 [DVDL – H]$^-$, 331 [gall-glc – H]$^-$, 313 [gall-glc – H_2O (– 18) – H]$^-$, 301 [EA – H]$^-$, 275 [LHHDP – CO_2 (– 44) – H]$^-$, 249 [HHBP – H]$^-$, 169 [GA – H]$^-$, 125 [GA – CO_2 (– 44) – H]$^-$	Cornusiin F (3)
19	4.92	465 [M – gall – H_2O (– 170) – H]$^-$, 331 [M – 2 x gall (– 304) – H]$^-$, 313 [M – 2 x gall – H_2O (– 322) – H]$^-$, 169 [GA – H]$^-$, 125 [GA – CO_2 (– 44) – H]$^-$	Tri-*O*-galloyl-β-D-glucose (2)
20	5.08	633 [M – gall (– 152) – H]$^-$, 301 [EA – H]$^-$, 275 [LHHDP – CO_2 (– 44) – H]$^-$, 249 [HHBP – H]$^-$, 169 [GA – H]$^-$, 125 [GA – CO_2 (– 44) – H]$^-$	Tellimagrandin I (2)
21	5.16	1085 [crn B – H]$^-$, 935 [M – cmp A (– 1418) – H]$^-$, 785 [tell I – H]$^-$, 765 [oen C – H_2O – H]$^-$, 633 [gall-HHDP-glc = gm D – H]$^-$, 613 [oen C – gall (– 152) – H_2O – H]$^-$, 451 [VTL – H]$^-$, 425 [DVDL – H]$^-$, 331 [gall-glc – H]$^-$, 313 [gall-glc – H_2O (– 18) – H]$^-$, 301 [EA – H]$^-$, 275 [LHHDP – CO_2 (– 44) – H]$^-$, 249 [HHBP – H]$^-$, 169 [GA – H]$^-$	Cornusiin C (1)
22	5.22	933 [M – gall (– 152) – H]$^-$, 783 [oen C = M – HHDP (– 302) – H]$^-$, 765 [oen C – H_2O (– 18) –H]$^-$, 451 [VTL – H]$^-$, 301 [EA – H]$^-$, 275 [LHHDP – CO_2 (– 44) – H]$^-$, 249 [HHBP – H]$^-$, 169 [GA – H]$^-$, 125 [GA – CO_2 (– 44) – H]$^-$	Cornusiin B
23	5.32	1417 [cmp A = M – gall (– 152) – H]$^-$, 935 [M – HHDP – gall – glc = gm D (– 634) – H]$^-$, 785 [tell I – H]$^-$, 783 [oen C = M – HHDP – 2 x gall – glc (– 786) – H]$^-$, 765 [oen C – H_2O (– 18) – H]$^-$, 633 [gall-HHDP-glc = gm D – H]$^-$, 451 [VTL – H]$^-$, 425 [DVDL – H]$^-$, 313 [gall-glc – H_2O (– 18) – H]$^-$, 301 [EA – H]$^-$, 275 [LHHDP – CO_2 (– 44) – H]$^-$, 249 [HHBP – H]$^-$, 169 [GA – H]$^-$, 125 [GA – CO_2 (– 44) – H]$^-$	Cornusiin A (2)
24	5.40	935 [M – HHDP – gall – glc = gm D (– 634) – H]$^-$, 785 [tell I – H]$^-$, 783 [oen C = M – HHDP – 2 x gall – glc (– 786) – H]$^-$, 765 [oen C – H_2O (– 18) – H]$^-$, 633 [gall-HHDP-glc = gm D – H]$^-$, 451 [VTL – H]$^-$, 425 [DVDL – H]$^-$, 313 [gall-glc – H_2O (– 18) – H]$^-$, 301 [EA – H]$^-$, 275 [LHHDP – CO_2 (– 44) – H]$^-$, 249 [HHBP – H]$^-$, 169 [GA – H]$^-$, 125 [GA – CO_2 (– 44) – H]$^-$	Cornusiin A (3)
25	5.58	935 [M – HHDP – gall – glc = gm D (– 634) – H]$^-$, 785 [tell I – H]$^-$, 783 [oen C = M – HHDP – 2 x gall – glc (– 786) – H]$^-$, 765 [oen C – H_2O (– 18) – H]$^-$, 633 [gall-HHDP-glc = gm D – H]$^-$, 451 [VTL – H]$^-$, 425 [DVDL – H]$^-$, 313 [gall-glc – H_2O (– 18) – H]$^-$, 301 [EA – H]$^-$, 275 [LHHDP – CO_2 (– 44) – H]$^-$, 249 [HHBP – H]$^-$, 169 [GA – H]$^-$, 125 [GA – CO_2 (– 44) – H]$^-$	Cornusiin A (4)
26	5.69	1085 [crn B – H]$^-$, 935 [M – cmp A (– 1418) – H]$^-$, 785 [tell I – H]$^-$, 765 [oen C – H_2O – H]$^-$, 633 [gall-HHDP-glc = gm D – H]$^-$, 613 [oen C – gall (– 152) - H_2O – H]$^-$, 451 [VTL – H]$^-$, 425 [DVDL – H]$^-$, 331 [gall-glc – H]$^-$, 313 [gall-glc – H_2O (– 18) – H]$^-$, 301 [EA – H]$^-$, 275 [LHHDP – CO_2 (– 44) – H]$^-$, 249 [HHBP – H]$^-$, 169 [GA – H]$^-$	Cornusiin C (2)
27	5.87	1085 [crn B – H]$^-$, 935 [M – cmp A (– 1418) – H]$^-$, 785 [tell I – H]$^-$, 765 [oen C – H_2O – H]$^-$, 633 [gall-HHDP-glc = gm D – H]$^-$, 613 [oen C – gall (– 152) – H_2O – H]$^-$, 451 [VTL – H]$^-$, 425 [DVDL – H]$^-$, 331 [gall-glc – H]$^-$, 313 [gall-glc – H_2O (– 18) – H]$^-$, 301 [EA – H]$^-$, 275 [LHHDP – CO_2 (– 44) – H]$^-$, 249 [HHBP – H]$^-$, 169 [GA – H]$^-$	Cornusiin C (3)
28	6.04	935 [M – HHDP – gall – glc = gm D (– 634) – H]$^-$, 785 [tell I – H]$^-$, 783 [oen C = M – HHDP – 2 x gall – glc (– 786) – H]$^-$, 765 [oen C – H_2O (– 18) – H]$^-$,	Cornusiin A (5)

		633 [gall-HHDP-glc = gm D – H]$^-$, 451 [VTL – H]$^-$, 425 [DVDL – H]$^-$, 313 [gall-glc – H_2O (– 18) – H]$^-$, 301 [EA – H]$^-$, 275 [LHHDP – CO_2 (– 44) – H]$^-$, 249 [HHBP – H]$^-$, 169 [GA – H]$^-$, 125 [GA – CO_2 (– 44) – H]$^-$	
29	6.15	1569 [M-gall (– 152) – H]$^-$, 1087 [M – gall-HHDP-glc = gm D (– 634) – H]$^-$, 1085 [crn B = M – 3 x gall – glc (– 636) – H]$^-$, 935 [M – HHDP – 2 x gall - glc = tell I (– 786) – H]$^-$, 785 [tell I – H]$^-$, 765 [oen C – H_2O (– 18) = M – 768 – 152 – 18 – H]$^-$, 633 [gall-HHDP-glc = gm D – H]$^-$, 451 [VTL – H]$^-$, 425 [DVDL – H]$^-$, 313 [gall-glc – H_2O (– 18) – H]$^-$, 301 [EA – H]$^-$, 275 [LHHDP – CO_2 (– 44) – H]$^-$, 249 [HHBP – H]$^-$, 169 [GA – H]$^-$, 125 [GA – CO_2 (– 44) – H]$^-$	Cornusiin D or Camptothin B (1)
30	6.34	1569 [M-gall (– 152) – H]$^-$, 1087 [M – gall-HHDP-glc = gm D (– 634) – H]$^-$, 1085 [crn B = M – 3 x gall-glc (– 636) – H]$^-$, 935 [M – HHDP – 2 x gall-glc = tell I (– 786) – H]$^-$, 785 [tell I – H]$^-$, 765 [oen C – H_2O (– 18) – H]$^-$, 633 [gall-HHDP-glc = gm D – H]$^-$, 451 [VTL – H]$^-$, 425 [DVDL – H]$^-$, 313 [gall-glc – H_2O (– 18) – H]$^-$, 301 [EA – H]$^-$, 275 [LHHDP – CO_2 (– 44) – H]$^-$, 249 [HHBP – H]$^-$, 169 [GA – H]$^-$, 125 [GA – CO_2 (– 44) – H]$^-$	Cornusiin D or Camptothin B (2)
31	6.49	785 [M – gall (– 152) – H]$^-$, 633 [gall-HHDP-glc = gm D = M – 2 x gall (– 304) – H]$^-$, 465 [M – HHDP – gall – H_2O (– 472) – H]$^-$, 447 [M – HHDP – gall – 2 x H_2O (– 490) – H]$^-$, 313 [gall-glc – H_2O – H]$^-$, 301 [EA – H]$^-$, 295 [M – HHDP – 2 x gall – 2 x H_2O (– 642) – H]$^-$, 275 [LHHDP – CO_2 (– 44) – H]$^-$, 249 [HHBP – H]$^-$, 169 [GA – H]$^-$, 125 [GA – CO_2 (– 44) – H]$^-$	Tellimagrandin II
32	6.69	617 [M – gall – H_2O (– 170) – H]$^-$, 465 [M – 2 x gall – H_2O (– 322) – H]$^-$, 447 [M – 2 x gall – 2 x H_2O (– 340) – H]$^-$, 313 [gall-glc – H_2O = M – 3 x gall – H_2O (– 474) – H]$^-$, 295 [gall-glc – 2 x H_2O = M – 3 x gall – 2 x H_2O (– 492) – H]$^-$, 169 [GA – H]$^-$, 125 [GA – CO_2 (– 44) – H]$^-$	Tetra-*O*-galloyl-β-D-glucose
33	6.78	275 [LHHDP – CO_2 (– 44) – H]$^-$, 249 [HHBP – H]$^-$	Ellagic acid
34	6.96	935 [M – cmp A – gall (– 1570) – H]$^-$, 785 [M – cmp A – HHDP (– 1720) = tell I – H]$^-$, 765 [oen C – H_2O – H]$^-$, 633 [gall-HHDP-glc – H]$^-$, 451 [VTL – H]$^-$, 313 [gall-glc – H_2O (– 18) – H]$^-$, 301 [EA – H]$^-$, 275 [LHHDP – CO_2 (– 44) – H]$^-$, 249 [HHBP – H]$^-$, 169 [GA – H]$^-$, 125 [GA – CO_2 (– 44) – H]$^-$	Trapanin A (β or α)
35	7.04	935 [M-tell I (– 786) – H]$^-$, 785 [tell I – H]$^-$, 765 [oen C – H_2O = M – tell II – H_2O (– 956) – H]$^-$, 633 [gall-HHDP-glc = gm D – H]$^-$, 451 [VTL – H]$^-$, 301 [EA – H]$^-$, 275 [LHHDP – CO_2 (– 44) – H]$^-$, 249 [HHBP – H]$^-$, 169 [GA – H]$^-$, 125 [GA – CO_2 (– 44) – H]$^-$	Cornusiin D or Camptothin B (3)
36	7.42	787 [M – gall (– 152) – H]$^-$, 769 [M – gall – H_2O (– 170) – H]$^-$, 617 [M – 2 x gall – H_2O (– 322) – H]$^-$, 465 [M – 3 x gall – H_2O (– 474) – H]$^-$, 295 [M – 4 x gall – 2 x H_2O (– 644) – H]$^-$, 169 [GA – H]$^-$, 125 [GA – CO_2 (– 44) – H]$^-$	Penta-*O*-galloyl-β-D-glucose (1)
37	7.77	787 [M – gall (– 152) – H]$^-$, 617 [M – 2 x gall – H_2O (– 322) – H]$^-$, 465 [M – 3 x gall – H_2O (– 474) – H]$^-$, 313 [M – 4 x gall – H_2O (– 626) – H]$^-$, 169 [GA – H]$^-$, 125 [GA – CO_2 (– 44) – H]$^-$	Penta-*O*-galloyl-β-D-glucose (2)

t_R, retention time; **MS²**, the second mass spectrum (fragment ions); **cmp A**, camptothin A; **crn B**, cornusiin B; **DVDL**, decarboxylated valoneic acid dilactone; **EA**, ellagic acid; **GA**, gallic acid; **gall**, galloyl; **glc**, β-D-glucose; **gm D**, gemin D; **HHBP**, hexahydroxybiphenyl; **HHDP**, hexahydroxydiphenoyl; **icr F**, isocoriariin F; **irg B**, isorugosin B; **LHHDP**, hexahydroxydiphenic acid monolactone; **oen C**, oenothein C; **tell I**, tellimagrandin I; **tell II**, tellimagrandin II; **VTL**, valoneic acid trilactone; main signals are underline
